# Early treatment of complex located pediatric low‐grade gliomas using iodine‐125 brachytherapy alone or in combination with microsurgery

**DOI:** 10.1002/cam4.605

**Published:** 2015-12-29

**Authors:** Mathias Kunz, Silke B. Nachbichler, Lorenz Ertl, Gunther Fesl, Rupert Egensperger, Maximilian Niyazi, Irene Schmid, Joerg Christian Tonn, Aurelia Peraud, Friedrich Wilhelm Kreth

**Affiliations:** ^1^ Department of NeurosurgeryLudwig‐Maximilians‐UniversityMunichGermany; ^2^ Department of Radiation OncologyLudwig‐Maximilians‐UniversityMunichGermany; ^3^ Department of NeuroradiologyLudwig‐Maximilians‐UniversityMunichGermany; ^4^ Center for Neuropathology and Prion ResearchLudwig‐Maximilians‐UniversityMunichGermany; ^5^ Department of Pediatric Oncology and HematologyLudwig‐Maximilians‐UniversityMunichGermany

**Keywords:** Best safe resection, complex tumor location, functional outcome, low‐dose rate I‐125 SBT, pediatric low‐grade glioma

## Abstract

To analyze efficacy, functional outcome, and treatment toxicity of low‐dose rate I‐125 brachytherapy (SBT) alone or in combination with best safe resection (in case of larger tumor volumes) as first‐line treatment for pediatric low‐grade gliomas (PLGGs) not suitable for complete resection. Consecutively treated (2000–2014) complex located circumscribed WHO grade I/II PLGGs were included. For small tumors (≤4 cm in diameter) SBT alone was performed; for larger tumors best safe resection and subsequent SBT was chosen. Temporary Iodine‐125 seeds were used (median reference dose: 54 Gy). Treatment response was estimated with the modified MacDonald criteria. Analysis of functional outcome included ophthalmological, endocrinological and neurological evaluation. Survival was analyzed with the Kaplan–Meier method. Prognostic factors were obtained from proportional hazards models. Toxicity was categorized according to the Common Terminology Criteria for Adverse Events. Fifty‐eight patients were included treated either with SBT alone (*n* = 39) or with SBT plus microsurgery (*n* = 19). Five‐year progression‐free survival was 87%. Two patients had died due to tumor progression. Among survivors, improvement/stabilization/deterioration of functional deficits was seen in 20/14/5 patients, respectively. Complete/partial response had beneficial impact on functional scores (*P* = 0.02). The 5‐year estimated risk to receive adjuvant radiotherapy/chemotherapy was 5.2%. The overall early (delayed) toxicity rate was 8.6% (10.3%), respectively. No permanent morbidity occurred. In complex located PLGGs, early SBT alone or combined with best safe resection preserves/improves functional scores and results in tumor control rates usually achieved with complete resection. Long‐term analysis is necessary for confirmation of these results.

## Introduction

Pediatric low‐grade gliomas (PLGGs) exhibit molecular genetic alterations different from that of the adult population. Clinically, PLGGs are generally characterized by slow growth rates, a low risk of malignant transformation, and excellent long‐term survival rates 1, 2, 3, 4, 5. Surgery is regarded the cornerstone of treatment: For those harboring completely resectable tumors, long‐term progression‐free survival (PFS) can be expected after surgery alone. In case of incompletely resectable or unresectable tumors, however, PFS becomes significantly shorter leading to the initiation of adjuvant treatment concepts including chemotherapy or radiotherapy relatively early during the history of the disease 6, 7, 8. Given the non‐negligible risk of short‐ and long‐term side effects of adjuvant treatment concepts currently in use 1, 6, 9, 10, 11, 12, ongoing interest exists to further expand the platform of localized treatment concepts for PLGG patients. A previously published pilot study has suggested that low‐dose rate stereotactic Iodine‐125 brachytherapy (SBT) allowing precise application of ablative tumor doses with maximum sparing of surrounding normal tissues might contribute to the spectrum of localized treatment concepts for complex located PLGGs; in that study SBT was used either alone in case of small‐sized unresectable tumors or coupled with best safe resection (in case of larger tumor volumes) and has turned out to be feasible and safe 13. A small sample size and short follow‐up periods, however, were limitations of this study. For further clarification this study was conducted. Stereotactic Iodine‐125 brachytherapy (SBT) alone or in combination with surgery was stringently used as front‐line therapy. Stereotactic Iodine‐125 brachytherapy (SBT) was preferred for complex located small‐sized tumors, whereas best safe resection plus subsequent SBT of the residual tumor was used for larger (diameter >4 cm) complex located PLGGs. Besides traditional endpoints such as PFS, we focused on functional outcome and side effects of the therapy. It was hypothesized that early SBT – either alone or in combination with microsurgery – would be safe and enhance treatment efficacy for those who would otherwise had received biopsy only or partial tumor resection.

## Methods

### Patients

**Figure 1:**
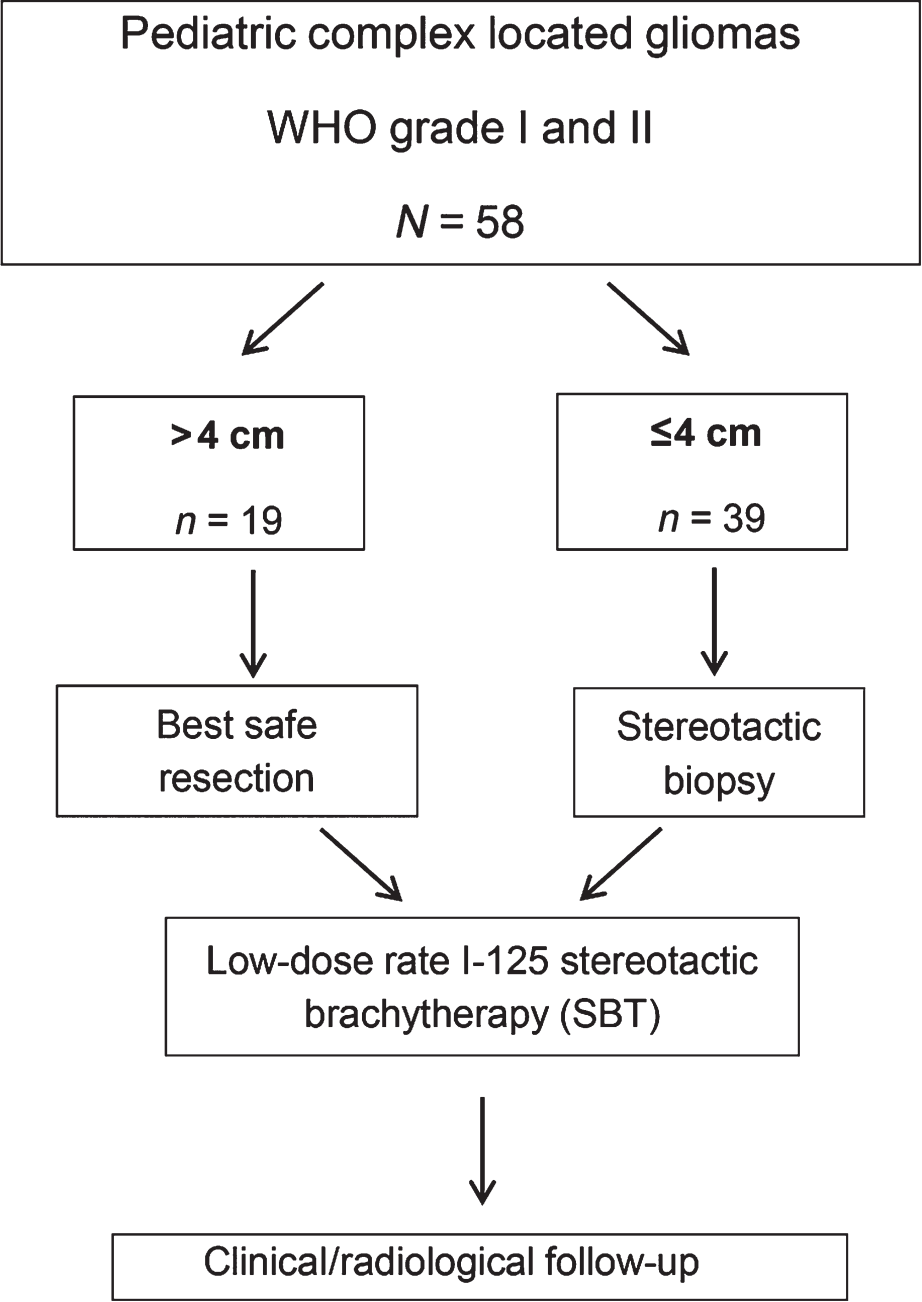
Study design.

Histologically proven circumscribed and complex located WHO grade I or II PLGGs (<18 years of age) not suitable for gross total resection were considered eligible for this study. Patients were consecutively enrolled between April 2000 and April 2014. Tumor classification was performed according to the WHO criteria 14. Patients data of the pilot study were updated and also included 13. Deep seated nonlobar located supratentorial tumors (including those infiltrating the insula of Reil), lobar located tumors involving functionally relevant areas (such as the primary motoric cortex or language‐related areas), and infratentorial tumors involving the brainstem or the cerebellar peduncle were classified as complex located tumors. Circumscribed tumors were those exhibiting nearly identical tumor volumes on both T1‐ and T2‐ magnetic resonance imaging (MRI) sequences. Before study inclusion, all tumors had to be classified as either incompletely resectable or unresectable. “Resectability” had been assessed by two experienced neurosurgeons (J. C. T, A. P) in the context of an interdisciplinary tumorboard. Tailored treatment concepts were applied prospectively according to the study protocol of the pilot study: 13 Stereotactic Iodine‐125 brachytherapy (SBT) alone was indicated in circumscribed tumors with a maximum diameter of 4 cm. Best safe resection was initiated for larger tumors with a diameter of more than 4 cm; SBT of the residual tumor was initiated 10–12 weeks after microsurgery (Fig. 1). All patients were required to have measurable disease on MRI. All patients undergoing SBT only received stereotactic biopsy for histological and molecular‐genetic characterization as described previously 15, 16. Stereotactic Iodine‐125 brachytherapy (SBT) was performed immediately after biopsy if the intraoperative evaluation indicated grade I/II histology. In uncertain cases, the result of paraffin embedded tissue examination was awaited and SBT was done 5–7 days later. A small subset of patients (*n* = 5) had received chemotherapy before study inclusion which was not considered an exclusion criterion. No patients had undergone initial radiotherapy. Informed consent was obtained from the parents or legal guardians and whenever possible from the patients themselves. The study protocol was reviewed and approved by the institutional review board (UE Nr. 131–14).

### Best safe resection

Neuronavigation, intraoperative ultrasound, and intraoperative neurophysiological monitoring/mapping techniques were used as described previously 13, 17, 18, 19. The anticipated extent of resection was preoperatively determined by both, two experienced neurosurgeons (J. C. T, A. P) and the stereotactic neurosurgeon (F. W. K.). It was aimed to define a threshold volume suitable for subsequent SBT.

### SBT protocol

**Figure 2:**
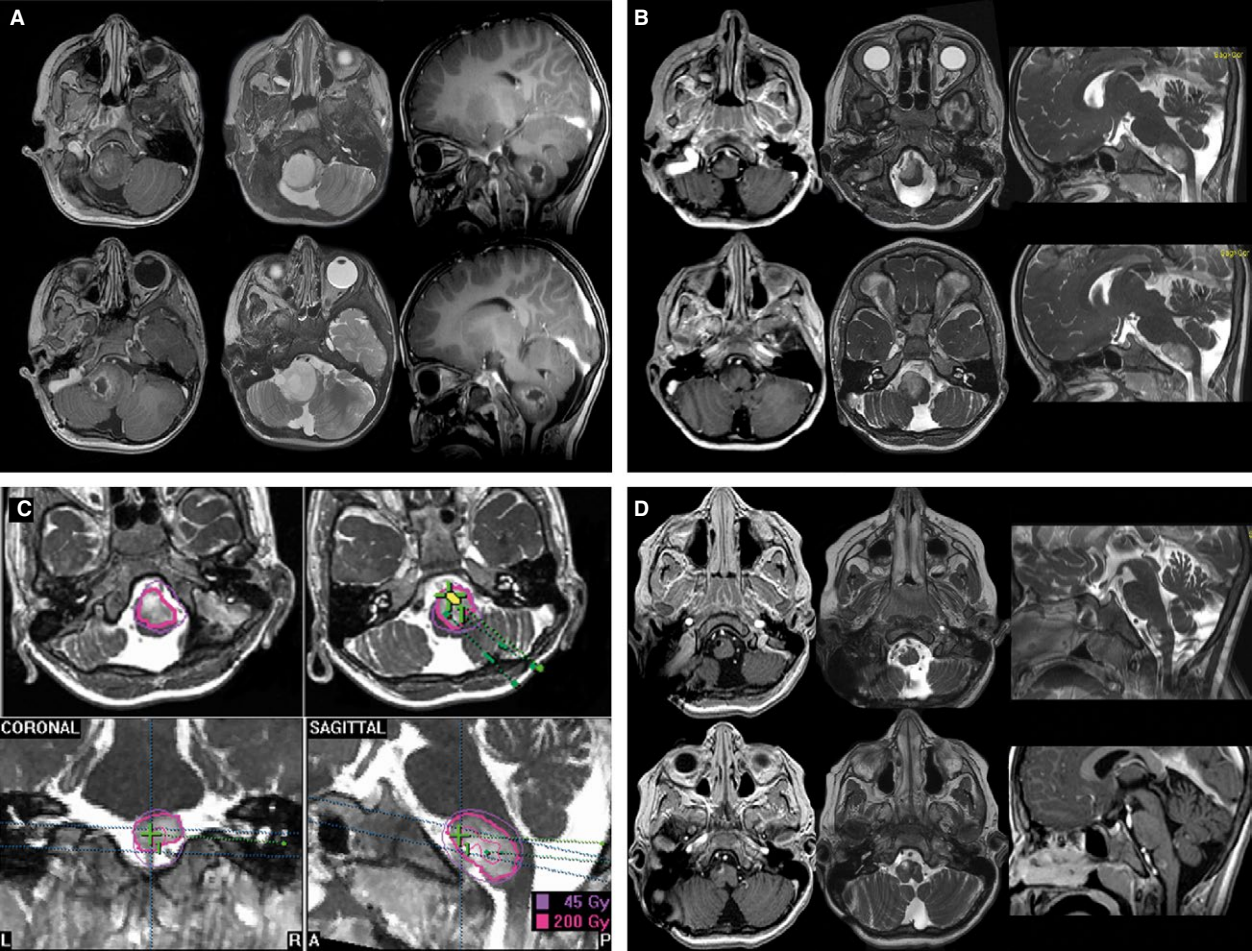
Selected treatment example indicating a child with a WHO grade I astrocytoma of the brainstem with exophytic growth undergoing combined treatment; (A) before treatment, (B) after best safe resection, (C) Iodine‐125 stereotactic brachytherapy (SBT) irradiation planning (three seeds), (D) 18 months post SBT showing partial response (PR).

**Figure 3:**
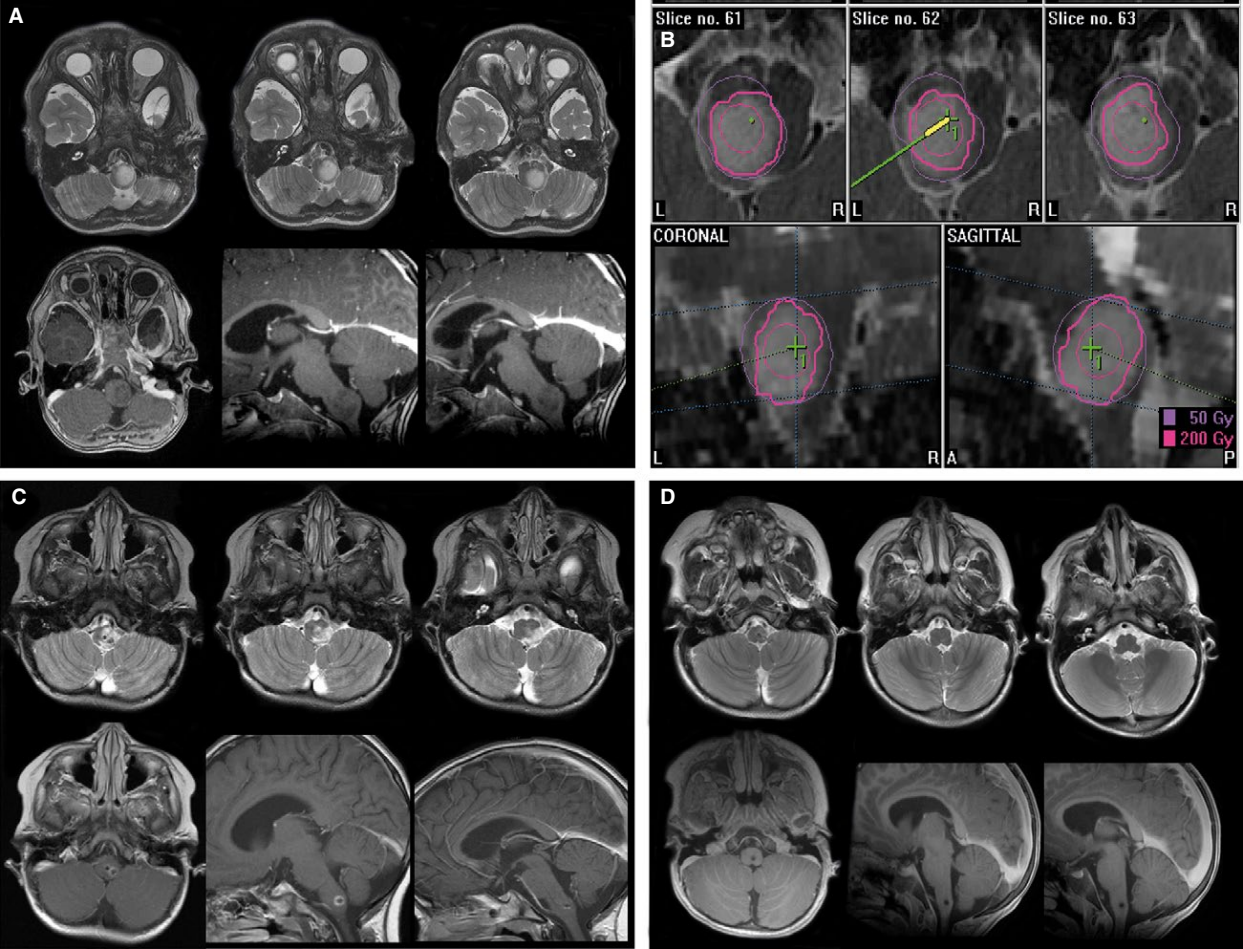
Selected treatment example indicating a child exhibiting a circumscribed WHO grade II glioma of the brainstem undergoing Iodine‐125 stereotactic brachytherapy (SBT) alone (A) before SBT, (B) SBT irradiation planning (one seed), (C) 6 months and (D) 12 months after SBT showing complete response (CR).

**Figure 4:**
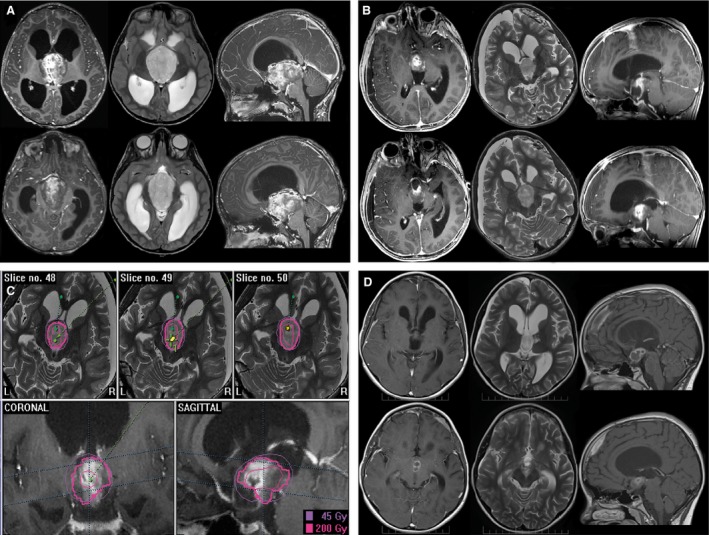
Selected treatment example indicating a child exhibiting a WHO grade I astrocytoma of the hypothalamic area undergoing combined treatment (A) before treatment, (B) after best safe resection, (C) Iodine‐125 stereotactic brachytherapy (SBT) irradiation planning (two seeds), (D) 6 months after SBT showing partial response.

Stereotactic Iodine‐125 brachytherapy (SBT) was done as described previously 13, 20, 21. Briefly, 3D‐treatment planning was performed with the @target software program (Brainlab, Feldkirchen, Germany) on the basis of colocalized CT‐ and MRI‐data. Low‐activity temporary Iodine‐125 seeds (<20 mCi) were exclusively used. Tumors were treated with a reference dose (calculated to the outer rim of the tumor) of 54 Gray (Gy); the dose rate was low (≤12 cGy/h). Treatment parameters were in line with those of numerous prospective and retrospective reports 21, 22, 23. In case of larger tumor volumes the reference dose was adjusted accordingly (50 Gy or even 45 Gy). Conformal irradiation of complex located tumors was achieved by implantation of multiple radioactive sources (e.g., 3–4 seed catheters). Representative examples are given in Figure 2, 3, 4.

### Molecular genetic profiling

From 2006 onwards molecular‐genetic analyses were stringently performed as described previously. The O^6^‐methylguanine‐DNA methyltransferase (*MGMT*) promoter methylation status was assessed by methylation‐specific polymerase chain reaction (PCR) and bisulfite sequencing; 24 for the determination of loss of heterozygosity (*LOH*) on *1p/19q* status microsatellite analysis was used; 25 both, isocitrate dehydrogenases (*IDH1/2*) and *BRAF* ^*V600E*^ mutational status were assessed by pyrosequencing 26, 27. For the determination of the *BRAF‐KIAA1549* fusion transcripts PCR was used 28.

### Patient evaluation

Clinical and neuroradiological examination were performed preoperatively, within 3 days after tumor resection, 3 months after SBT and then in 6 months intervals. Magnetic resonance imaging (MRI) was performed on 1.5T or 3.0T scanners (Magnetom Symphony, Siemens; or Signa HDxt, GE Healthcare, WI, USA). The standardized protocol comprised an axial T2‐weighted sequence (slice thickness: 2 mm) and 3D T1‐weighted sequences (slice thickness: 1 mm) before and after administration of gadopentetate dimeglumine (0.1 mmol per kilogram of body weight [Magnevist, Schering] Berlin, Germany). Preoperative tumor volume was defined by outlining the contour of the lesion on each slice of the respective MRI sequence. Volume calculation was done using the @target software program. Postoperative follow‐up MR investigations sometimes slightly differed from the preoperative protocol in terms of slice thickness and were partially performed in an outpatient setting. Treatment response was assessed according to the modified MacDonald criteria for low‐grade gliomas 29. This was done at the time of last follow‐up for those without tumor progression. In those suffering from tumor progression over time the MRI before progression was used for assessment of the best treatment response. Tumor size after SBT was determined by the product of the two largest perpendicular diameters of the hyperintense T2 lesion in nonenhancing tumors. In enhancing tumors response criteria considered both the size of the hyperintense T2 lesion and any changes of the enhanced T1‐weighted images. Pseudoprogression was assumed when contrast enhanced areas after SBT transiently increased and resolved thereafter. Treatment toxicity was classified according to the Common Terminology Criteria for Adverse Events (CTCAE), Version 4.0 NIH, 2010. Acute toxicities were identified as events that arose within 90 days of the start of SBT and late toxicities as events that occurred thereafter. All MRI scans were independently reviewed by two experienced neuroradiologists (L. E, G. F). In case of discordant findings a conference for reevaluation was initiated to achieve a consensus concerning the final radiological evaluation. In case of suspected tumor progression stereotactic biopsy or open surgery was performed to verify tumor progression/malignant transformation.

For performance analyses before treatment and at the time of last follow‐up the Lansky score 30 was assessed. Evaluation of functional scores before treatment and at follow‐up investigations included motor, visual, endocrine function and seizure activity; it was independently performed by the attending pediatric physicians, ophthalmologists, and endocrinologists. The current analysis referred to functional parameters at the time of last follow‐up for those exhibiting no tumor progression. For those exhibiting tumor progression last findings before tumor progression were used. Deficits in motor function were distributed into mild (muscle strength grade 4–5 according to the British Medical Research Council grading system, mild ataxia) and severe (muscle strength ≤4, severe ataxia). Functional outcome was classified as improved (complete disappearance of a mild motor deficit, improvement from a severe to a mild deficit), deteriorated (worsening from a mild to a severe motor deficit, appearance of a new motor deficit) and unchanged (stabilized motor deficit). Ophthalmological tests included visual acuity measurements, perimetry of the visual field and oculomotor function. Evaluation was performed in children exhibiting tumors in the hypothalamic/suprasellar location, the thalamus and basal ganglia (optic tract), the brainstem, the cerebellar peduncle, and in tumors of the occipital or temporomesial lobe. Deficits in visual acuity were distributed into mild (0.9–0.5) and severe (0.4–0), visual field deficits into partial anopsia and complete hemianopsia and oculomotor function into mild (incomplete affection of one oculomotor nerve) and severe (complete affection of one or more oculomotor nerves) deficits. Functional outcome was classified as improved (increased visual acuity of ≥0.2, decreased mean defect according to visual field perimetry, complete remission of mild or improvement of severe oculomotor deficits), deteriorated (decreased visual acuity of ≥0.2, increased mean defect, worsening of oculomotor function from mild to severe or corresponding new deficits); otherwise visual function was classified as unchanged. In hypothalamic/suprasellar tumor location endocrinological evaluation included basal serum levels of growth hormone GH, Insulin‐like‐growth factor IGF‐1, ACTH, baseline cortisol, prolactin, luteinizing and follicle stimulating hormone LH and FSH, thyreotropine TSH and thyroid hormone, testosterone, and estradiol. Neurohypophyseal function was determined by serum sodium and osmolarity level, fluid intake and output and urine specific gravity level. Endocrinological deficits were pre and postoperatively categorized as anterior or posterior hypopituitarism. This classification concerned both those with complete and incomplete insufficiency of the affected lobe. In case of involvement of both lobes (partial or complete), insufficiency was classified as panhypopituitarism. Postoperative improvement was assumed in case of both complete recovery of at least one affected hormonal axis and at least unchanged findings in the remaining axes. Any new complete or partial insufficiency of a single hormonal axis 6 weeks after surgery was classified as deterioration. Otherwise the endocrinological status was classified as unchanged.

Disappearance of seizures (with/without anticonvulsive drugs) was classified as improvement. An increased seizure activity (including frequency and/or semiology) or a new onset of seizures at the time of last follow‐up was classified as deterioration. Otherwise a stabilized unchanged seizure status was assumed.

### Statistical analysis

The reference point of the study was the date of seed implantation. Date of last follow‐up was April 2015. Primary endpoints were tumor progression, functional outcome, and treatment toxicity. Continuously scaled variables were described as median (mean) including the range of their distribution. Patient‐ and tumor‐related variables were analyzed and compared with the chi‐squared statistics (for dichotomized variables) and the Wilcoxon test (for continuously scaled variables); Wilcoxon test for related samples was used to analyze changes of functional variables over time. Time to tumor progression and time to malignant transformation were analyzed with the Kaplan–Meier method. Survival curves were compared with the log‐rank test. Prognostic factors were obtained from proportional hazards models. A *P*‐value ≤0.05 was considered significant. All calculations were performed using the SAS software package version 9.2 Heidelberg, Germany.

## Results

The study population comprised 58 patients (23 female, 35 male) with a median age of 9 years (median follow‐up: 49 months [12–162]). Thirteen patients were younger than 5 years. Five patients had progressive tumors after pervious chemotherapy. Those with recurrent tumors are not different form the others in terms of histology, age, Lansky score, and tumor size. WHO grade I tumors were found in 43 patients. Seven patients were suffered from neurofibromatosis type I (NF‐1). Patients with grade II tumors had better initial Lansky scores (*P* = 0.04) and more often lobar located tumors (*P* = 0.004). Shunt placement before tumor treatment was done in six patients. Patients′ characteristics are summarized in Table 1. *MGMT* promoter methylation (2/24 tumors), mutated *IDH 1/2* status (1/23), or *LOH 1p/19q* codeletion (2/23) were seldom found, whereas a *BRAF* ^V600E^ mutation/presence of *BRAF‐KIAA1549* fusion transcripts were frequently seen in WHO grade I tumors (10/16).

**Table 1:** Patients’ characteristics (*n* = 58)

Characteristics			
Therapy	*n* (%)	Treatment parameters of SBT Median[range]	*P* Value
Microsurgery + SBT	19 (32.8%)	54 Gy, 11.3 cGy/h [10–18], 450h*	*n.s.
SBT	39 (67.2%)	54 Gy, 9.4 cGy/h [4–15], 600h*	

	Overall	Microsurgery + SBT	SBT	*P* Value
Age (years) Median [range]	9 [0.9–17]	8 [1.5–16]	11 [0.9–17]	n.s.
Sex				
Female	23 (40%)	3 (16%)	20 (51%)	0.02
Male	35 (60%)	16 (84%)	19 (49%)	
Lansky score Median [range]	90 [60–100]	90 [60–100]	90 [60–100]	n.s.
Histology				
WHO I	43 (74%)	16 (84%)	27 (69%)	n.s
WHO II	15 (26%)	3 (16%)	12 (31%)	
Location				
Lobar	11 (19%)	5 (26%)	6 (15.5%)	n.s.
Hypothalamic/suprasellar	16 (28%)	9 (47%)	7 (18%)	0.03
Thalamus/basal ganglia	15 (26%)	0^a^	15 (38.5%)^c^	(a)+(b) versus (c)+(d)
Brainstem	9 (15%)	2 (11%)^b^	7 (18%)^d^	**0.001**
Cerebellum (pedunculus)	7 (12%)	3 (16%)	4 (10%)	n.s.
Tumor volume (ccm) Median [range]				(e) versus (f) (e) versus (g)
Before microsurgery	8.7 [0.4–88]	26 [3.5–88]^a^	–	**0.001**
Before SBT	4.8 [0.4–21]	4.8 [0.4–19]^b^	5.1 [0.7–21]^c^	(f) versus (g) n.s.

WHO, World Health Organization; SBT, Iodine‐125 stereotactic brachytherapy; Gy, Gray; h, hours; n.s., not significant.

Thirty‐nine patients underwent SBT alone and 19 patients a combined treatment. The initial tumor volume was 26 ccm in the combined group and 5.1 ccm in the SBT only group (*P* = 0.001); this difference was no longer present after best tumor resection (4.8 ccm vs. 5.1 ccm). Tumors of the hypothalamic region were more often seen in the combined group (*P* = 0.03), whereas tumors of the thalamus/basal ganglia and the brainstem predominantly occurred in the SBT only group (*P* = 0.001). The SBT only and the combined group did not differ in terms of age, Lansky score, tumor type and the frequency of preoperative functional deficits (Table 1). Seeds have been removed after a median time interval of 550 h [220–1200] after implantation in an outpatient manner; no complications have been observed.

### Treatment response

**Figure 5:**
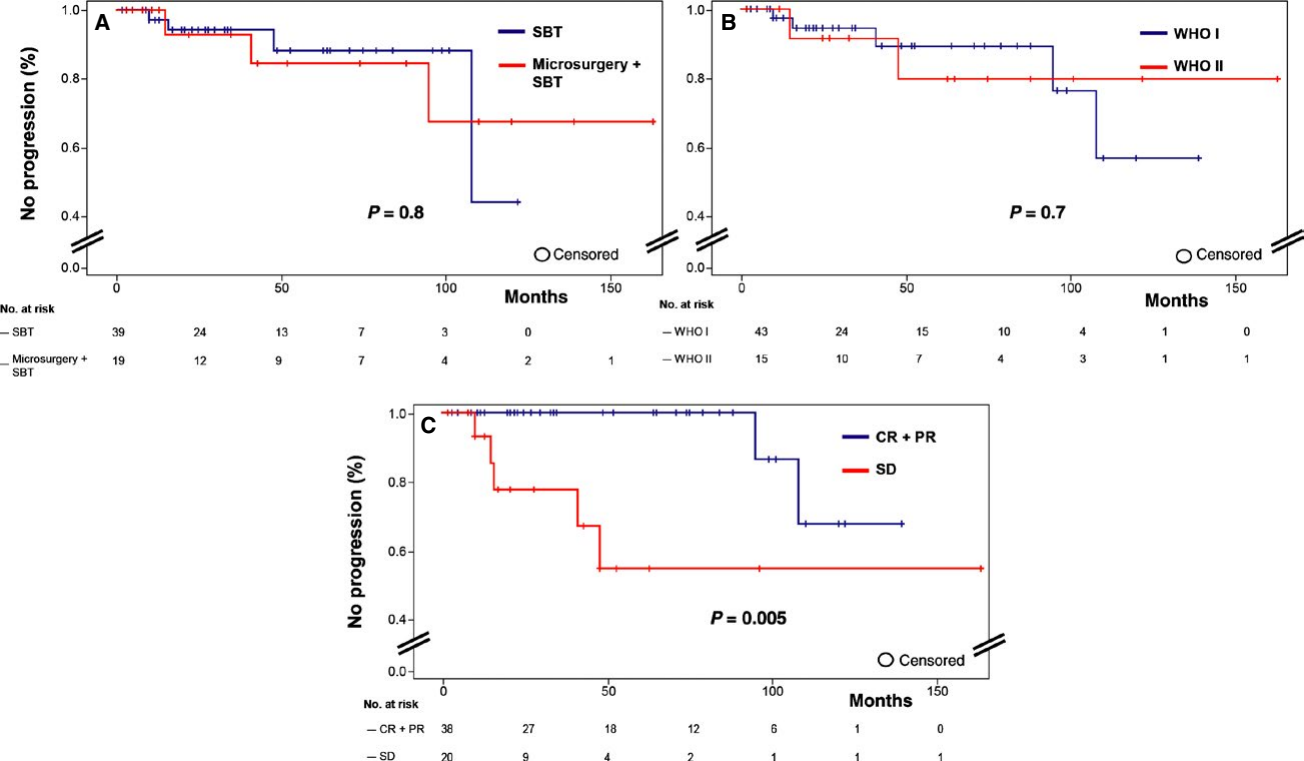
Kaplan–Meier curves of progression‐free survival (PFS) stratified by (A) treatment modality (stereotactic low‐dose rate Iodine‐125 brachytherapy (SBT) only versus combined with best safe microsurgical resection), (B) WHO grade I versus II and (C) overall best treatment response (complete response (CR) + partial response (PR) versus stable disease (SD)).

Best treatment response was seen after a median time of 16 months after SBT; complete response (CR), partial response (PR), and stable disease (SD) occurred in 10, 28, and 20 patients, respectively (Fig. 2, 3, 4 display treatment examples). Seven patients (12%) exhibited biopsy proven progressive disease (PD) including one patient with malignant transformation of a previous WHO grade II astrocytoma. Response rates were identical in both treatment groups and not influenced by the NF‐1 status. Two patients had died due to tumor progression. One of these children suffered from a malignant transformation of an initial astrocytoma WHO grade II 24 months after initial treatment. Overall, 5‐year progression‐free survival (PFS) and 5‐year overall survival (OS) were 87% (95% CI: 69.8–94.5) and 95% (95% CI: 81.9–98.8), respectively. Favorable factors for PFS were CR/PR (*P* = 0.005) and a Lansky score ≥90 (*P* = 0.02); tumors with a *BRAF* ^V600E^ mutation/*BRAF‐KIAA1549* fusion transcripts (10/16) showed more often CR/PR (8/10 vs. 1/6; *P* = 0.04). WHO grade (*P* = 0.7), tumor location (*P* = 0.5), tumor size (*P* = 0.2), NF‐1 status (*P* = 0.6) and the applied tailored treatment strategy (*P* = 0.8) did not gain prognostic influence. Progression‐free survival (PFS) is detailed in Figure 5. Two children, harboring grade I gliomas, experienced late tumor progression 95 and 108 months after initial treatment. One patient has received combined treatment and the other one SBT alone. *BRAF^V600E^* mutation was seen in one patient. Progression was proven by biopsy in one patient, who underwent subsequent Re‐SBT. The other underwent open tumor resection. Histological reevaluation revealed a grade I tumor recurrence. Salvage treatment after progression consisted of resection alone (*n* = 3), resection plus external beam irradiation (*n* = 1), SBT retreatment (*n* = 1), chemotherapy (*n* = 1), or combined radiochemotherapy (*n* = 1). The 5‐year estimated risk to receive additional radiotherapy/chemotherapy was 5.2%.

### Functional outcome

At the time of last follow‐up, the median Lansky score of the survivors (*n* = 56) was 90 [60–100]; the differences between the respective performance scores preoperatively and at last follow‐up were statistically not significant (*P* = 0.7). Functional outcome was not influenced by the mode of treatment. Deficits were initially seen in 41 patients and occurred significantly more often in case of a hypothalamic tumor location (*P* = 0.003). Those children without functional deficits preoperatively (*n* = 17) remained all asymptomatic at the time of last follow‐up. In surviving patients initially exhibiting functional deficits (*n* = 39) improvement, stabilization, and deterioration were seen in 20, 14, and 5, respectively. Functional improvement occurred more often after CR or PR compared to those with SD (*P* = 0.02). Functional deterioration was more often observed in those suffering from PD; the difference, however, was statistically not significant. In detail, improvement concerned 9/13 motor‐, 11/19 visual‐ (visual acuity, perimetry, and oculomotor function), 3/14 endocrine‐, and 6/6 seizure‐associated deficits; deterioration concerned 1/13 motor‐ and 2/14 endocrine‐associated deficits; new functional postoperative deficits included oculomotor disturbance and endocrine deficits in respective one patient (Table 2). Improvement concerned more often motor/visual/seizure‐associated deficits whereas endocrine function often remained unchanged or even worsened (*P* = 0.04).

**Table 2:** Functional outcome

Preoperative deficits	N (%)	Postoperative outcome at last F/U			
		Improvement	Stabilization	Deterioration	New deficit
Motor					
Mild	7	5	2	0	0
Severe	6	4	1	1	0
Total	13 (22)	9 (69.2)	3 (23.1)	1 (7.7)	0
Visual					
Visual acuity					
Mild	6	3	3	0	0
Severe	4	2	2	0	0
Visual field disturbances					
Partial deficit	4	3	1	0	0
Hemianopsia	3	2	1	0	0
Oculomotor disturbances					
Mild	1	1	0	0	0
Severe	1	0	1	0	1
Total	19 (33)	11 (57.9)	8 (42.1)	0	1 (1.8)
Endocrinological function					
Anterior hypopituitarism	7	1	5	1	1
Posterior hypopituitarism	3	1	2	0	0
Pan‐hypopituitarism	4	1	2	1	0
Total	14 (24)	3 (21.4)	9 (64.3)	2 (14.3)	1 (1.8)
Seizure activity	6 (10)	6 (100)*	0	0	0
		With/without anticonvulsive drugs (4/2)			

### Treatment toxicity

The overall early toxicity rate was 8.6%. Early grade 1 and 2 toxicities have been observed in 1 and 3 patients, respectively (transient deterioration of a preexisting hemiparesis). Early grade 3 toxicity was seen in one patient (progressive hydrocephalus demanding shunt surgery).

**Figure 6:**
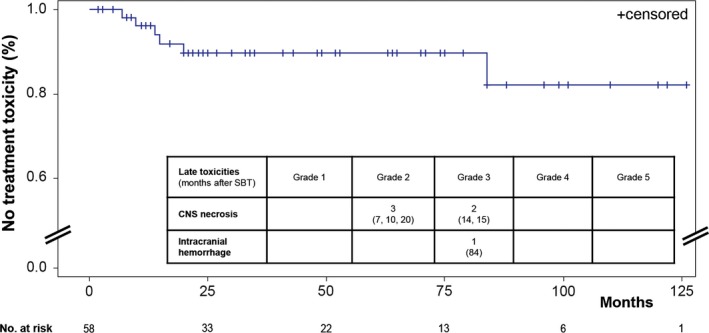
Kaplan–Meier curve indicating late treatment toxicity after Iodine‐125 stereotactic brachytherapy (SBT) with respective detailed toxicities shown.

The overall late toxicity rate was 10.3% (grade 2: 3 patients, grade 3: 3 patients; Fig. 6). Most events (83%) were seen within the first 2 years after SBT. The estimated 1‐ and 2–year risk of late treatment toxicity was 6.4% and 10.3%, respectively. Patients suffering from late treatment toxicity had significantly larger tumor volumes (11.1 ccm vs. 4.5 ccm; *P* = 0.02). Complications were manageable in all patients and did not result in permanent morbidity: three patients were transiently treated with steroids (median treatment time: 21 days); in two patients surgical treatment with necrosectomy became necessary; additionally, one patient suffered from an intralesional bleeding of a blister aneurysm of the internal carotid artery 7 years after implantation requiring surgical occlusion. Ad hoc analysis of the dose distribution indicated that the high‐dose zone (200 Gy isodose) had included parts of the internal carotid artery.

### Symptomatic pseudocyst formation after SBT

Symptomatic space‐occupying pseudocyst formations were seen in 11 patients (19%). The estimated 1‐ and 2–year risk for symptomatic pseudocysts was 9 and 23%, respectively. Clinical symptoms completely resolved after stereotactically guided drainage in all cases (internal shunt (6)/stereotactic aspiration (5)); no risk factor could be identified.

## Discussion

The role of SBT for initial treatment of PLGGs is still under debate. In a recently published multicenter treatment study for PLGGs (HIT‐LGG–1996), for example, SBT has been classified as a mode of radiation therapy and was therefore used in a delayed fashion for selected small‐sized progressive tumors 6. Stereotactic Iodine‐125 brachytherapy (SBT), however, integrates biological characteristics of both radiosurgery and fractionated external beam radiation: The application of a highly localized intratumoral necrotizing dose in the vicinity of the radioactive source (high‐dose zone) goes hand in hand with protective effects of fractionation particularly in the microenvironment of the interface between normal brain and neoplastic cells 21, 22, 31. Given the highly localized treatment effects of SBT, it is tempting to use SBT – in analogy to surgical treatment – as primary treatment modality for complex located PLGGs. This approach was tested in our previously published pilot study^13^ and is now further explored in the current report. Given the fact that tumor volume restrictions exist for SBT 20, 22, a tailored surgical treatment concept was tested: Small‐sized complex located PLGGs were treated by SBT alone, whereas larger tumors underwent best safe resection and SBT of the residual tumor thereafter. It was hypothesized that the early use of SBT alone or in combination with best safe resection offers the possibility to treat the entire tumor volume in complex located PLGGs. It is important to note that SBT was used as upfront treatment strategy in order to avoid/withhold adjuvant treatment such as chemotherapy/conventional radiotherapy, or new molecular based therapies currently under investigation. The somewhat unique radiobiological characteristics of SBT explain that long‐term radiogenic complications are generally not expected to occur and that the treatment spectrum is not narrowed by early SBT. In other words, fractionated radiotherapy still remains a valuable and safe treatment option for progressive tumors after previously performed SBT 20, 22, 23, 32.

This is to our knowledge the first study, which systematically analyzed SBT alone or in combination with microsurgery (in case of larger tumors) as an early treatment concept for pediatric patients with unresectable, complex located gliomas under consideration of a detailed functional outcome analysis. We here demonstrate that both SBT alone and combined treatment of PLGGs is associated with PFS rates as long as that reported after complete resection in more recent studies (5–year PFS: 87% in the current series vs. approximately 90%) 6, 7, 8, 33. Furthermore, SBT alone as compared to microsurgery (in combination with SBT) for larger tumors has been shown to be similar effective. Prognostic evaluation revealed favorable influence of a Lansky score ≥90. The presence of *BRAF* ^V600E^ mutation/*BRAF‐KIAA1549* fusion was more often associated with CR/PR. The impact of this biomarker on PFS has been recently reported 12, 34, 35.

The results of the current report could not be easily compared with that of other studies dealing with SBT in PLGGs. These studies had relied in parts on CT‐data alone for treatment planning and response evaluation, performed SBT inconsistently sometimes early or delayed, and did not systematically analyze combined concepts for larger tumors 23, 32.

Whether the early use of SBT alone or in combination with best safe resection might attract a higher efficacy than delayed SBT remains unknown and deserves further prospective evaluation. The impact of initial treatment, however, should not be underestimated: Initial complete resection has turned out to be one of the strongest favorable prognostic factors (for PFS and OS) in the HIT‐LGG‐1996 study supporting a concept of early radical surgical treatment; unfortunately, complete resection was only possible in 359 out of 1031 patients mostly due to highly eloquent tumor locations 6. We here show that the applied tailored treatment concept enables treatment of the entire tumor volume (as depicted on the MRI) in complex located unresectable or only partial resectable PLLGs. The chosen approach overcomes limitations typically associated with SBT (i.e., tumor size) or microsurgical treatment (i.e., eloquent tumor location); patients harboring larger tumors or deep seated ones did not do worse in terms of PFS in the current series which contrasts finding of recently published reports 6, 23.

Studies on functional deficits after treatment are extremely scarce. We here show that the applied tailored treatment strategy could either improve or stabilize functional deficits in the overwhelming number of patients (87%). Functional recovery/stabilization rates were not different in both treatment groups. We found associations between treatment response and functional outcome: those undergoing complete/partial response did significantly better than those exhibiting tumor control. Seventeen patients were asymptomatic at the time of treatment and considered to be at risk of functional deterioration due to their complex tumor location. All these patients continued to be asymptomatic at the time of last follow‐up supporting our concept of early treatment. The preservation/improvement of function in the majority of patients was in line with the hypothesized protective effects of both SBT and best safe resection. The provided data might be regarded as a basis against which other functional outcome data of future studies could be compared. However, long‐term analysis is necessary to confirm these study results.

We further show that chemotherapy and/or external beam radiation could be withheld in the vast majority of patients including 13 patients younger than 5 years of age; this subpopulation has been shown to be in particular vulnerable to the effects of external beam radiation 9. The estimated risk for receiving adjuvant treatment was nearly identical with that reported after complete resection; it strongly contrasts risk profiles for adjuvant treatment after partial resection or biopsy only (estimated 5–year risk for adjuvant treatment after subtotal resection: approximately 45%; after partial resection/biopsy only: approximately 75%) 6.

Ideally, expected long‐term survival should go hand in hand with diminishing of long‐term treatment‐related complications. We observed a blister aneurysmal bleeding of the internal carotid artery 7 years after treatment in one patient. Re‐analysis of the radiation plan indicated high‐dose radiation in the area of the occurring aneurysm. Even though other studies have reported on occlusive arteriopathies after high‐dose radiation 36, the risk of a radiation‐induced aneurysm has not been acknowledged so far. Given the extreme heterogeneity of the radiation field of SBT and the strong interrelation between both vessels and the tumor particularly in case of suprasellar tumors or those of the insula of Reil, high‐dose radiation of arteries is sometimes difficult to avoid. Unfortunately, analyses concerning threshold doses and the risk of SBT‐induced arteriopathies are still lacking. Given the results of this and other reports 36, however, one should be aware of such risks and try to avoid high‐dose radiation of arteries (>120 Gy) whenever possible.

The frequency of radiogenic complications occurring within the first 2 years after SBT was completely in line with other long‐term analyses dealing with SBT 20, 22. The current report further confirmed the tumor volume before SBT to be a relevant risk factor for complications. The median tumor volume of those exhibiting a radiogenic complication was 11.1 ccm. Given these results indications of SBT for larger tumor volumes should be critically evaluated concerning their potential benefit and their risk. The sample of this study, however, was not suitable to define a threshold volume beyond that radiogenic complications might occur.

Low‐grade gliomas can develop pseudocyst formations during the course of the disease. The impact of treatment on this phenomenon remains unclear. The observed frequency in the current report appeared to be in line with other reports 23, 32. Pseudocyst formations can be usually managed by stereotactic guided aspiration or implantation of an “internal” shunt catheter thereby immediately relieving clinical symptoms.

In summary, we provide evidence that tailored surgical treatment using either SBT alone or in combination with best safe resection extends the initial treatment platform for unresectable or incompletely resectable PLGGs: favorable risk profile, preservation of function, and PFS as long as after complete resection might be regarded as the hallmarks of this new treatment concept for complex located PLGGs.

## Conflict of Interest

No conflict of interest disclosures from any author.
